# An Adaptive Control Method for Ros-Drill Cellular Microinjector with Low-Resolution Encoder

**DOI:** 10.1155/2013/418068

**Published:** 2013-03-06

**Authors:** Zhenyu Zhang, Nejat Olgac

**Affiliations:** Department of Mechanical Engineering, ALARM Lab, University of Connecticut, Storrs, CT 06269, USA

## Abstract

A novel control methodology which uses a low-resolution encoder is presented for a cellular microinjection technology called the Ros-Drill (rotationally oscillating drill). It is developed primarily for ICSI (intracytoplasmic sperm injection) operations, with the objective of generating a desired oscillatory motion at the tip of a micro glass pipette. It is an inexpensive setup, which creates high-frequency (higher than 500 Hz) and small-amplitude (around 0.2 deg) rotational oscillations at the tip of an injection pipette. These rotational oscillations enable the pipette to drill into cell membranes with minimum biological damage. Such a motion control procedure presents no particular difficulty when it uses sufficiently precise motion sensors. However, size, costs, and accessibility of technology to the hardware components severely constrain the sensory capabilities. Consequently, the control mission and the trajectory tracking are adversely affected. This paper presents two contributions: (a) a dedicated novel adaptive feedback control method to achieve a satisfactory trajectory tracking capability. We demonstrate via experiments that the tracking of the harmonic rotational motion is achieved with desirable fidelity; (b) some important analytical features and related observations associated with the controlled harmonic motion which is created by the low-resolution feedback control structure.

## 1. Introduction and Motivation 

We provide some background on the main task at hand and relevant motivation, before the control methodology is elaborated. ICSI (intracytoplasmic sperm injection) is a broadly utilized technique for artificial fertilization. This procedure is successfully performed in human oocytes as well as other species such as mouse and bovine. First, a holding pipette is used to immobilize an individual oocyte with a slight suction. Then an injection pipette (with outer diameter of about 8 *μ*m), which contains the sperm head to be injected, is forced into the cell. The piercing through the zona layer and the membrane needs to be achieved with minimal biological damage to facilitate rapid healing. A significant amount of research effort has been devoted towards developing microscopic instruments for ICSI from this perspective alone. The most popular procedure at the present is the piezo-assisted ICSI [1]. However, its piercing performance is successful only by using a small mercury droplet in the pipette tip [2, 3]. Without this addition, undesirable lateral oscillations occur at the tip and severely hamper the performance of piercing. Due to high toxicity of mercury, on the other hand, the piezo-assisted ICSI procedure is forbidden in many biological laboratories. In recent years, an improved remedial technology, called rotationally oscillating drill (Ros-Drill), is introduced [4]. This technique shows comparable results to those obtained by the piezo-assisted ICSI process, with one major difference, that Ros-Drill does not have the mercury problem [5]. 

**Figure 1:**
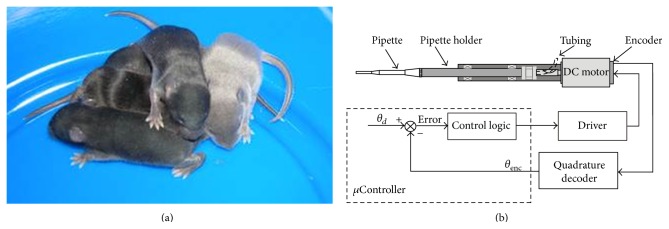
(a) Black mice reproduced using Ros-Drill technology (white mouse is the surrogate mother) [5]. (b) Ros-Drill assembly and control system.

A schematic of the Ros-Drill assembly is shown in Figure 1. The injection pipette is connected to a small-precision micromotor which is controlled to track a desired sinusoidal trajectory:

(1) $$\begin{array}{c} \theta_{d}=A_{d}\sin\left(2\pi f_{d}\cdot t\right), \end{array}$$

where *A*_*d*_ is the oscillation amplitude (typically around 0.2 deg), and *f*_*d*_ is the frequency (in the range of 500–700 Hz). These selections are based on a simple bandwidth analysis; at this frequency range of external stimuli the cell membrane is not expected to follow the pipette tip motion. The ensuing relative rotational motion between the pipette and the cell membrane creates a clean piercing action, which facilitates the rapid healing of membrane after piercing. 

Considerable amount of experimental effort has been invested to demonstrate the validity of Ros-Drill technology [4]. It is shown that the success rate in ICSI by Ros-Drill is comparable to that of piezo-assisted technology, provided that the pipette oscillations are maintained as close as possible to the desired trajectory in (1). Many healthy mouse offspring are produced using the Ros-Drill methodology, as shown in Figure 1(a). These biological tests are conducted by a group of experimentalists from the University of Connecticut and the University of California, Davis, USA. Two outstanding requirements are noted in these experiments towards an acceptable Ros-Drill performance: (i) rotational oscillation to track the harmonic trajectory very closely; (ii) the flexible pipette to be concentric to the rotational axis of Ros-Drill. The later condition is shown to satisfy because of the extreme bending compliance and whirling effects [2, 3]. The condition (i) is the topic of this paper. The smooth variation of the rotational motion in accordance with a harmonic function is the most natural desired trajectory. The contribution of this paper is a proper control law which can serve the objective under severe sensing constraints. Overarching restriction in this study comes from the pricing aspect. In order to make the Ros-Drill accepted by a wide range of IVF clinics, the cost of this automated device must remain within $1500 per copy; otherwise, it would be difficult to compete against the commonly-used but inefficient method adopted by trained ICSI specialists. These conditions set the tone of the key research issues.

Let us take a closer look at the physics of the Ros-Drill microinjection procedure. The rotational stiffness of the pipette holder and the pipette including the extremely fine tip are assumed to be high. Consequently, the angular displacement, *θ*, of the injection pipette is transmitted from the shaft of the micromotor to the pipette tip without a loss. The lateral (bending) vibrations are the major concern at the tip, and the present operating scheme is intended to suppress them to ignorable levels especially when compared with those oscillations caused by the piezo-assisted ICSI [2, 3]. With these assumptions, the major objective of this study is directed to insure desirably precise harmonic motion tracking capabilities at the pipette tip [6–8] despite the very coarse sensory capabilities.

For position servoing, in general, the sensors are expected to have high resolution vis-à-vis the range of the intended motion, which can yield a desirable tracking capability. For instance, in representing a harmonic trajectory, one expects to have a minimum of 10 discrete data points per cycle. However, resolution of digital encoders is limited by the number of slots on a rotating disk, through which the encoder's light beam travels. Although advances in encoder technology have wonderfully progressed to increase the number of slots in order to improve the resolution of the encoders, the trade-off between resolution and cost is unavoidable. In such applications as Ros-Drill where the cost limitations are very stringent, sensor resolution is often compromised. 

We encounter considerable past research on control methods using some low-resolution sensors. Recent model-based speed observers [9] make the velocity estimate robust using an interesting disturbance observer. In another effort, Kwon et al. [10] incorporate acceleration measurement in velocity estimation and motion control. Bautista-Quintero and Pont [11] propose an H-infinity control algorithm for sensor-constrained mechatronic systems using the position sensors with relatively low resolutions. They demonstrate how this procedure allows a faithful reproduction of observed motion starting from a limited sensing ability using relatively common (and inexpensive) microcontrollers. Furthermore, the methods which deal with control applications with low-resolution sensors usually have high computational demands to compensate the sensory shortfall. This aspect quickly makes the method prohibitive in a cost-sensitive design such as Ros-Drill.

We wish to familiarize the reader with the components of the first-generation Ros-Drill setup and the current improvements. The first-generation design having a 512 lines/revolution-encoder with quadrature signature is the finest selection we could find within the cost and spatial confines. Its resolution is 0.17 deg (including the quadrature signature feature). It allows a maximum of 2-step reading over the desired peak-to-peak stroke (note that the desired amplitude is 0.2 deg) [4, 12]. This sensor makes our best observation of a Ros-Drill harmonic cycle with a 2-step representation, which is a colossal handicap to perform the control. The spirit of the proposed control scheme and the focus of this paper are primarily on this crucial feature.

Moving on to another component, the first trial generation of Ros-Drill employs a PLC (programmable logic controller) as its digital controller, which has a maximum sampling speed of 1 KHz. This constraint clearly limits the maximum frequency of the controlled trajectory to 500 Hz. Most recently reported ICSI tests use injection pipette oscillations up to 0.3 deg amplitude and maximum frequency of 500 Hz. These oscillations last a certain length of time which we name the *duration of oscillation*, *D*. Typically, *D* varies within 250–500 msec. The preliminary reports claim that Ros-Drill-assisted ICSI results in embryo survival, embryo development, birth, and weaning rates comparable to those of piezo-assisted ICSI using mercury [5]. Although these biological results are very promising, the trajectory tracking performance of the existing prototype is not satisfactory mainly because of the low resolution of position sensor and low control sampling rate of PLC. This work represents an effort to further improve this performance.

 In the second-generation (current) Ros-Drill microinjector prototypes, the low-sampling-frequency matter is considerably improved by replacing the PLC with a microcontroller (which brings the sampling speed from 1000 Hz to 10000 Hz) and the encoder resolution issue by selecting one-level higher-capacity sensor (1000 lines and 0.09 degree resolution after quadrature [12]). However, despite this upgrade, the position sensor with the resolution of 0.09 degrees still presents the biggest hurdle in this control system design because the required harmonic motion of 0.2 degree amplitude displays only 4-step peak-to-peak encoder recording, which is still low. In [12], a look-up-table-based adaptively tuned PID control law is used to cope with the limitations in the hardware. The most appropriate PID (proportional, integral, and derivative) control gains are a priori selected corresponding to the given operating frequency using dynamic simulations. For a given desired trajectory, *θ*_*d*_, with *A*_*d*_ and *f*_*d*_ attributes, an ad hoc 3D search routine is performed using the representative SIMULINK simulation program. This yields a set of feedback gains, which are then utilized through a look-up adaptation structure. They are regarded as nominal control gains as they are tuned based on the nominal model of Ros-Drill, and the control design is considered as the first adaptation stage towards the second-generation Ros-Drill.

An important point to discuss for this first adaptation stage is that the look-up-table-based control law can handle the potential parameter uncertainties, disturbances, and the unmodeled mechanical properties of the system only via the supervisory interference from the user. These uncertainties are important and unavoidable. For instance, the external load originating from the membrane resistance torque may vary with the type of species and it may affect the control performance of the system. Still worse is the extremely stringent requirement of concentricity on the shaft of motor and pipette holder via a coupling and ensuing resistance to the servosystem. In order to achieve better updates of the control gains, we introduce a second adaptation scheme in this paper. This procedure constitutes the primary contribution of the paper. Furthermore, the low-resolution encoder is a range indicator (as discussed later) rather than a measurement device. We offer some novel observations on the actual angular motion. 

The text is organized as follows. In the first part of the paper, a dedicated novel adaptive feedback control method is presented to achieve a satisfactory trajectory tracking capability with poor-quality sensors. Some important definitions about encoder signal are listed in Section 2. The first adaptation scheme is revisited in Section 3. In Section 4, the second-stage adaptive law is developed to update the control gains in situ, which is validated by experiments. In the second part of the paper, a stochastic perspective is presented which correlates the encoder readings with the actual angular motion.  Section 5 presents an intriguing observation over the trajectory tracking process. Finally, conclusions are given in Section 6. 

## 2. Some Descriptors of the Encoder Signal

**Figure 2:**
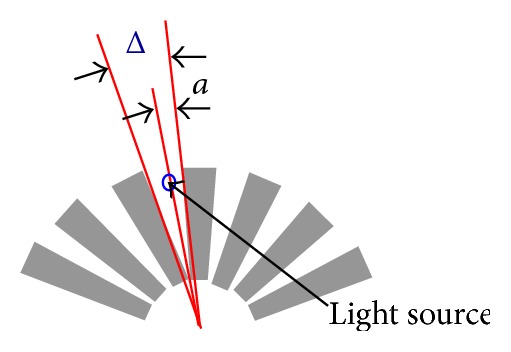
Encoder schematic and functionalities.

The highlighted feature in this study is the unusually low-resolution sensor (encoder).  Figure 2 gives a description of the operating principle of an optical encoder which is our main sensor. For simplicity, we overlook the quadrature effect and depict an encoder disk as the combination of transparent and dark lines. Δ is the angular resolution of the encoder. The small circle indicates the position of light beam. The angular motion *θ* denotes the rotational motion of the encoder. At the start, the encoder is declared at its “zero position” and *θ* = 0. 

We now present a critical argument on the position detection ability of the optical encoder. It is about an encoder offset angle, which we will denote by *a*, 0 < *a* < Δ. It is the angular motion needed for the first encoder pulse to register under a counterclockwise rotation (see Figure 2). “*a*” can be taken as a dead zone during which the encoder does not respond. Notice that this offset angle is not really an “offset position” from a baseline configuration. On the contrary, it is a measurement where we observe the first pulse. This value presents no importance at all, when the monitored motion is a few orders of magnitude larger than the resolution, Δ. However, for the particular application here the complete range of motion is composed of only a few Δ's (e.g., 4Δ). Thus, the offset angle *a* plays a very critical role and we present a set of novel observations on this issue, later in the paper.

**Figure 3:**
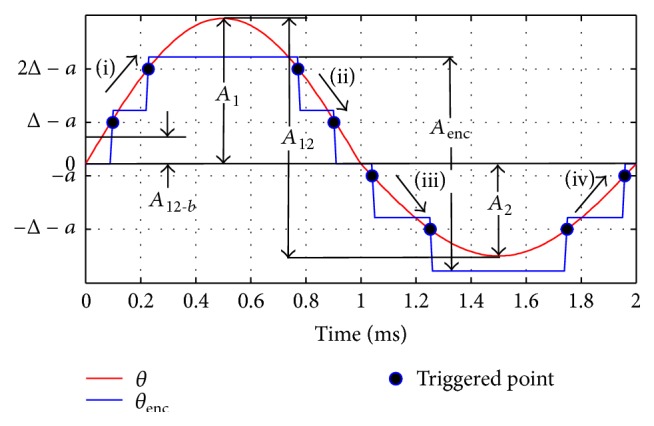
Oscillatory motion *θ* read by encoder (blue discrete lines represent the encoder signal); 0 < *a* < Δ is the random offset angle.

Clearly, *a* is an unknown quantity which is random and uniformly distributed within 0 < *a* < Δ. That is, from the starting position *θ* = 0 the encoder does not register any reading until −*a* degree of rotation (in counterclockwise sense) or −*a* + Δ degree (in clockwise sense) is completed (see Figure 2). In Figure 3, we further depict the sensing ability of the encoder on a hypothetical oscillation *θ* (shown as the red line). The encoder can only register when *θ* reaches angular displacements of −*a* + Δ, −*a* + 2Δ,…, −*a* + *m*Δ clockwise and −*a*, −*a* − Δ,…, −*a* − *m*Δ counterclockwise. Therefore, the responses to the same angular motion may vary; that is, the encoder may sense the same *θ* differently, depending on encoder offset angle, *a*.

Of course, if the resolution of encoder is high compared to the total stroke, such an offset angle would not cause a noticeable difference in detecting the motion. We represent this behavior using a quantizer block in SIMULINK if the resolution is high. However, for low-resolution encoders (i.e., relative to the stroke), such a quantizer block cannot reveal the true response of the encoder, as depicted in Figure 3. For such cases, the correct model will be introduced in Section 3.2.

Some definitions are provided next, in Figure 3; *A*_1_ and *A*_2_ are the upper and lower amplitudes of *θ*, respectively. Without loss of generality, we assume (*A*_1_ > *A*_2_ > 0) and define the complete actual peak-to-peak stroke as *A*_12_ = *A*_1_ + *A*_2_. The blue line represents the rotational motion being sensed by the encoder and the encoder reading is denoted as *θ*_enc_. The peak-to-peak angular stroke which is recorded by the encoder is named *A*_enc_, which is an integer multiple of Δ (e.g., 4Δ in Figure 3). We denote the average of *A*_12_ and *A*_enc_ over a certain number of oscillations by $\bar{A_{12}}$ and $\bar{A_{\text{enc}}}$, respectively. The bias of actual angular motion is defined as the distance of center of *A*_12_ from “zero” and it is expressed as

(2) $$\begin{array}{c} A_{12\text{-}b}=\frac{A_{1}-A_{2}}{2}. \end{array}$$

For a certain frequency, the hypothetical actual oscillations of the pipette can be described by the bias and the amplitude of actual angular motion. Let us express *θ* as

(3) $$\begin{array}{c} \theta =A_{12\text{-}b}+A\sin\omega t, \end{array}$$

where *ω* = 2*πf* and *A* and *f* are amplitude and frequency of harmonic wave, respectively, and we have *A*_12_ = 2*A*. To summarize these definitions, we list them again as follows:

(i) resolution of position sensor = Δ;
(ii) encoder offset angle = *a* (deg);
(iii) upper stroke of actual angular position from rest = *A*_1_ > 0 (deg);
(iv) lower stroke of actual angular position from rest = *A*_2_ > 0 (deg);
(v) actual peak-to-peak angular stroke = *A*_12_ = *A*_1_ + *A*_2_ (deg);
(vi) bias of actual angular stroke = *A*_12-*b*_;
(vii) peak-to-peak angular stroke detected by the encoder = *A*_enc_ (deg);
(viii) average actual angular stroke over a certain number of oscillations = $\bar{A_{12}}$ (deg);
(ix) average encoder stroke over a certain number of oscillations = $\bar{A_{\text{enc}}}$ (deg).

## 3. Review of Earlier Work: The First-Stage Adaptation Scheme

This section presents a brief review of the earlier work [12], which establishes the departure points of the present effort.

### 3.1. Control Objective and the Sensitivity Analysis

In Ros-Drill application, the desired angular trajectory requires high control sampling frequency, *f*_*s*_, in the microcontroller (such as 10 KHz) in order to perform a meaningful tracking. This imposes some further constraints on the limited computational capabilities of selected microcontroller. Keeping these restrictions in mind, a proportional integral and derivative (PID) control logic is adopted here. 

The Ros-Drill microinjector can be considered as a simple rotational mass, which is attached to a torque-generating DC servomotor (Figure 1). This yields a transfer function of Ros-Drill system and the corresponding frequency response creates the magnification factor as a function of *ω*, *K*_*p*_, *K*_*i*_, and *K*_*d*_:

(4) $$\begin{array}{c} M=\left|G\left(s=j\omega \right)\right|=M\left(f_{d},K_{p},K_{i},K_{d}\right), \end{array}$$

where *ω* = 2*π* · *f*_*d*_ and *K*_*p*_, *K*_*i*_, and *K*_*d*_ are the control gains. The objective of the control is to achieve a flat response over a given range of operating frequencies. That is, *M*(*f*_*d*_, *K*_*p*_, *K*_*i*_, *K*_*d*_) ≈ 1. We wish to emphasize that the phase angle between the desired harmonic input and the resulting output is not considered as a part of the performance description. Another important point to stress is that since we have limited access to the actual rotationally oscillating motion via the coarse measurements of the encoder, this flat response characteristic can be achieved, at best, by enforcing the peak-to-peak strokes of the encoder readings to be equal to *A*_enc_ = floor (2*A*_*d*_/Δ) · Δ deg.

It is shown in [12] that *M* is much more sensitive to the *K*_*d*_ variations rather than *K*_*p*_ and *K*_*i*_. We compare the effects of variation of these three control gains around their nominal values (Δ*K*/*K*). This results in some percentage variation of magnification factor (Δ*M*/*M*). Among the three gains (*K*_*d*_, *K*_*p*_, and *K*_*i*_), the most effective one is *K*_*d*_, from this perspective. Thus, the tuning of the magnification factor can be achieved more directly by utilizing only *K*_*d*_ variations. This tuning methodology needs to be further adapted to the actual sample data system when using a low-resolution encoders. The logic steps of this tuning methodology, or the adaptive update laws of *K*_*d*_, form the main contributions of this study, and they will be detailed in the following sections.

### 3.2. SIMULINK Model

**Figure 4:**
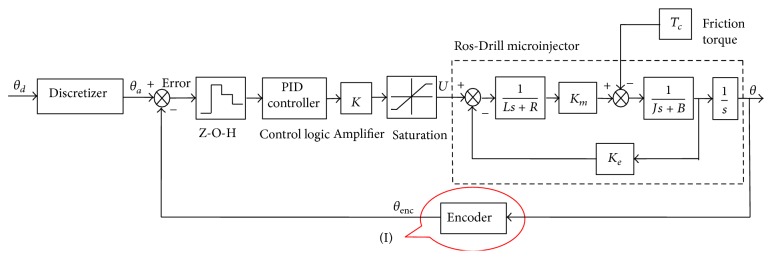
SIMULINK model of the spatially and temporally discrete control system.

In this section, we present a realistic dynamic model of the system using MATLAB-SIMULINK platform.  Figure 4 shows this model for the sample data PID control system. The encoder block is created to reflect the limitations of the position sensor as closely as possible to the reality. Two pieces of information are used from the quad signature encoder outputs: one is the angular step counter, and the other is the velocity of the angular motion. These data divide the angular movement space into four sections:

(5) $$\begin{array}{c} \left(\text{i}\right)\;\;\theta >0,\;\;\dot{\theta }>0\\ \left(\text{ii}\right)\;\;\theta >0,\;\;\dot{\theta }<0\\ \left(\text{iii}\right)\;\;\theta <0,\;\;\dot{\theta }<0\\ \left(\text{iv}\right)\;\;\theta <0,\;\;\dot{\theta }>0. \end{array}$$

**Figure 5:**
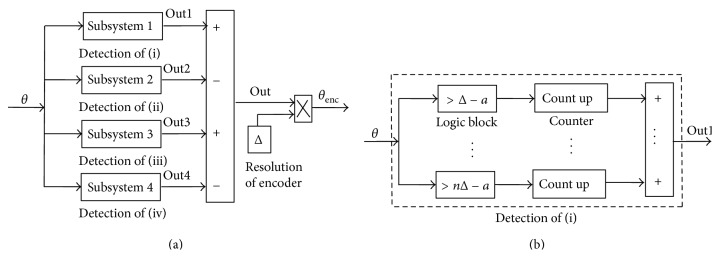
(a) Encoder reading scheme. (b) Model of the spatially discrete *θ*.

Notice that only one of these four cases will be active at any sampling period; thus, the summation of the four outputs will actually declare the one-step angular accumulation. The angular measurements, *A*_*d*_, in zero-order-hold (Z-O-H) mode is created with sampling time *T*_*s*_ just to simulate the sample data procedures of the microcontroller, see Figures 5(a) and 5(b). The integration step size for the simulation routine is chosen to be one-tenth of the sampling period of microcontroller (10 *μ*s), which is sufficient to represent the transient behavior within a sampling period with a desirable fidelity.

The amplifier gain, *K*, in Figure 4 is chosen by the user based on experimental knowledge such that the saturation in the D/A converter is prevented. The section enclosed by dotted lines in Figure 4 is the dynamic model for Ros-Drill microinjector itself. Using this SIMULINK tool, we first determine some starting values of the control gains *K*_*p*_, *K*_*i*_, and *K*_*d*_ using a pole-placement-based method as described in [12]. Then, we systematically vary *K*_*d*_. The objective in this tuning procedure is to obtain *M* = 1 as close as possible for the particular interval of frequencies. For a desired motion of *θ*_*d*_ = *A*_*d*_sin(2*π* *f*_*d*_ · *t*), *M* = 1 implies a peak-to-peak stroke of 2*A*_*d*_. This is the continuous rotational angle and the corresponding peak-to-peak encoder recording should be *A*_enc_ = floor (2*A*_*d*_/Δ) · Δ. 

We continue the systematic variations of *K*_*d*_ until the frequency response amplitude condition, *M* = 1, is achieved for the given operating frequency. We then repeat the same operation for other *f*_*d*_ values on a list of potential (i.e., biologically required) operating frequencies. This set of *K*_*d*_ gains will form a look-up table which can be used by the real-time control program on the microcontroller. Once the user identifies a preferred harmonic frequency, the program selects a *K*_*d*_ feedback gain from a given list and adaptively sets the new control logic. This completes the first adaptation scheme, which follows the steps below:

(a) declare the desired amplitude and frequency via GUI (*A*_*d*_, *f*_*d*_);
(b) select the corresponding control gain (*K*_*d*_) using the look-up table;
(c) observe the encoder registrations of peak-to-peak strokes, *A*_enc_;
(d) evaluate the average of *A*_enc_ over a predetermined duration *D*, $\bar{A_{\text{enc}}}$_;_
(e) signal the operator the direction of the error $e=\text{floor}\;(2A_{d}/\Delta )\cdot \Delta -\bar{A_{\text{enc}}}$ when it is outside a tolerance range, |*e*|> tolerance. Notice that *A*_*d*_ is typically not an integer multiple of Δ. Therefore, we deploy 2*A*_*d*_/Δ to create a comparable basis with $\bar{A_{\text{enc}}}$;
(f) manually adjust the gain, *K*_*d*_, as supervisory fine tuning and repeat the steps (c)–(f) until no violation of tolerance in (e) remains.

This procedure is shown to perform the task so that the peak-to-peak strokes of Ros-Drill are guaranteed to stay desirably close to *A*_enc_ = floor (2*A*_*d*_/Δ) · Δ [12]. 

### 3.3. Experimental Setup Used for All the Tests

**Figure 6:**
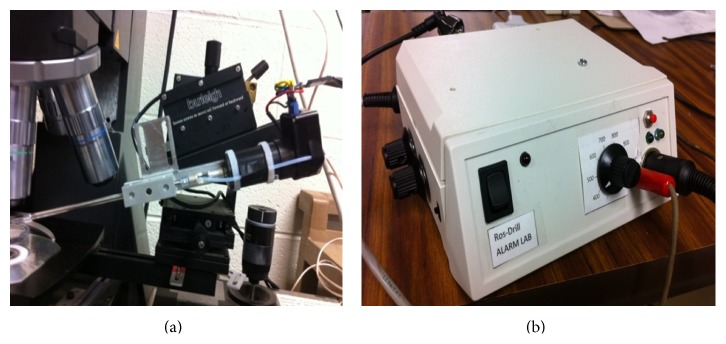
Experimental setup: (a) microinjector. (b) Control box.

One common setting was utilized during the different phases of progress.  Figure 6 shows that experimental setup, the mechanical device along with the controller box. A FAULHABER series 2342 micromotor is the actuator driving the pipette holder. An optical encoder with 1000 lines is utilized as position sensor, enabling the angular resolution at 0.09 deg (with quad-signature characteristics). It is important to clarify that this is the best option for an encoder in terms of size, resolution, and costs demanded by the application. A control box contains the components that handle the logical operations between the sensor (encoder) and the actuator (micromotor). Its main unit (the CPU and I/O device) is a Silicon Lab's C8051F121 *μ* controller. This box also contains the necessary peripheral circuits such as the converter of encoder's quadrature-signatures into the rotational pulse counter and direction determination of the rotation, optoisolators, and power output chip which feeds directly into the DC micromotor. 

We wish to give an idea to the reader about the microcontroller's time management structure. For the aimed Ros-Drill application, 10 KHz SISO (single input-single output) control sampling rate is selected, and the C8051F121 micro-controller can easily accommodate this speed. Out of this 100 *μ*s total loop time, 71.8 *μ*s is used for sensing, control logic evaluation, and D/A conversion; the remaining 28.9 *μ*s is the idle time which can be devoted to other applications (such as filtering) to be added later. 

In order to crossvalidate the capabilities of the tracking control, we use an independent angular motion monitoring tool. It is a dSPACE 1104 DSP card which simply performs encoder decoding duties. The motion validations provided in the entire text are from this sensing channel instead of the unit which handled the same task in the loop.

## 4. New Procedure: Second-Stage Adaptation Scheme

We present the main novelty of this paper in this section. The look-up-table-based control law offered in [12], as we summarized above, can handle the potential parameter uncertainties, disturbances, and the unmodeled mechanical properties of the system only via the supervisory interference from the user, see step (f) above. For a typical application such as Ros-Drill, these uncertainties originate mainly from friction-related sources, which are impossible to model accurately. We include them in our SIMULINK model (Figure 4) as some arbitrary combinations of viscous and coulomb friction effects, just to get a feel.

The control objective is again to achieve a sustained peak-to-peak stroke of *A*_enc_ = floor (2*A*_*d*_/Δ) · Δ as the encoder registers it (as an integer multiple of Δ). Remember that this is the only real-time measuring ability we are given. The average deviation of the *A*_enc_ (over a predetermined number of cycles) from this desired *A*_enc_ = floor (2*A*_*d*_/Δ) · Δ is used as the error for tuning of *K*_*d*_. One must pay attention, however, to another angle of these encoder readings. During the experiments, it is commonly observed that *A*_enc_ fluctuates typically between two successive steps (say 3Δ and 4Δ). This feature occurs due to the random offset angle *a* as mentioned above, but also stems from the transient regimes where the oscillations are not yet set into periodically repeated format. To discriminate the two causes of the same effect is an impossible task. Throughout this study, we consider the motion to have reached a steady regime for simplicity, therefore disqualifying the latter cause. The effects of the transient behavior are left for a future document. 

In this paper, we confine the execution of supervisory gain adjustments: (a) through a windowing and averaging procedure; (b) with sufficient update-and-wait period. In short, before we adaptively select *K*_*d*_ we allow sufficient length of oscillations to be recorded. Therefore, *K*_*d*_ can change only after a certain number of oscillations. In the ICSI experiment, typically entire duration of oscillations, *D*, lasts less than 500 ms. The tuning of *K*_*d*_ is expected to be completed within a small fraction of *D* and in the very early stages of the period *D*. After extensive tests using SIMULINK with additional disturbances imposed, it is observed that the angular position of the servo system (*θ*) reaches the steady state no later than 5 oscillations for all the operating frequencies (from 400 to 1000 Hz). Therefore, we use the average of peak-to-peak strokes within 15 cycles of oscillations, which we denote by  $\bar{A_{\text{enc}}}$, to assess the performance of current control gains and to update them. Obviously, if  $\bar{A_{\text{enc}}}$  is equal to *A*_enc_ = floor (2*A*_*d*_/Δ) · Δ that is considered to be satisfactory for the objectives of the control. 

In summary, this adaptation scheme follows the steps listed below:

(a,b,c) are identical to the first adaptation scheme above;
(d) evaluate the average of *A*_enc_ over 15 cycles, $\bar{A_{\text{enc}}}$;
(e) utilize an update law for *K*_*d*_ adjustments if the error, *e*, is outside a tolerance range, that is, |*e*|> tolerance. If the error is within the tolerance, no *K*_*d*_ update is needed.
(f) repeat step (d).

These steps will again guarantee the execution of the desired *A*_enc_ using an adaptive control gain update law. The details of this adaptation are given below. For the sake of simplicity, from this point onwards we will take *A*_*d*_ = 0.2 degrees and the corresponding *A*_enc_ = 4Δ = 0.36 degrees.

### 4.1. The Adaptive Update Law

Let us define *K*_*d*_(*n*) as the *n*th update of the derivative gain and *e*(*n*) as ensuing amplitude error which is obtained over 15 oscillatory cycles (an informed selection based on observation of experimental data), after *K*_*d*_(*n*) is applied. That is,

(6) $$\begin{array}{c} e\left(n\right)=4\Delta -\bar{A_{\text{enc}}}. \end{array}$$

For the next 15-cycle period, we use a new feedback gain with the following update law:

(7a) $$\begin{array}{c} K_{d}\left(n\right)=K_{d}\left(n-1\right)+\Delta K_{d}\left(n\right), \end{array}$$

where

(7b) $$\begin{array}{c} \Delta K_{d}\left(n\right)=C\cdot e\left(n-1\right), \end{array}$$

and *C* is an update constant. This update process is performed in the following sequences:

(i) start with *K*_*d*_(0) which is taken from a look-up table as explained in Section 3.
(ii) Determine the resulting *e*(0) at the end of the following 15 cycles.
(iii) For *n* = 1, use Δ*K*_*d*_(1) = *C* · *e*(0) and evaluate *K*_*d*_(1) from (7a).
(iv) Again after 15 cycles of using this control gain, determine *e*(1).
(v)
  (A) If |*e*(1)| ≤ |*e*(0)|, evaluate Δ*K*_*d*_(2) = *C* · *e*(1), *K*_*d*_(2) = *K*_*d*_(1) + Δ*K*_*d*_(2), assign *K*_*d*-ref_ = *K*_*d*_(1) and *e*_ref_ = *e*(1), and go to (vi).
  (B) If |*e*(1)| > |*e*(0)|, evaluate Δ*K*_*d*_(2) = 0.5 *C* · *e*(0), *K*_*d*_(2) = *K*_*d*_(0) + Δ*K*_*d*_(2) and determine *e*(2). If |*e*(2)| ≤ |*e*(0)|, assign *K*_*d*-ref_ = *K*_*d*_(2) and *e*_ref_ = *e*(2), go to (vi). If |*e*(2)| > |*e*(0)|, repeat (vB) but this time with Δ*K*_*d*_(3) = 0.5  ∗0.5*C* · *e*(0) and continue until the absolute error falls below *e*(0). Assign the current *K*_*d*_(*n*) value to *K*_*d*-ref_, current *e*(*n*) value to *e*_ref_  and move to (vi).
(vi) After 15 cycles, determine the new *e*(*n*) and go to (vA). Use comparison of |*e*(*n*)| ≤ |*e*_ref_|.

Some nuances on this gain adaptation process are discussed later in this section over an example data set along with the role of *K*_*d*-ref_ and *e*_ref_.

The following portion is devoted to the selection of the update constant *C*. Uncertain friction term is modeled as some combination (in SIMULINK model of Figure 4) of viscous and coulomb frictions:

(8) $$\begin{array}{c} T_{f}\left(t\right)=T_{c}\cdot \mathrm{sgn}\left(\dot{\theta }\right)+B\cdot \dot{\theta }, \end{array}$$

where *T*_*c*_ is the coulomb friction torque and *B* is the viscous friction coefficient. Note that different friction scenarios refer to the model in Figure 4 with the same system parameters but with combination of different *T*_*c*_ and *B*. Scenarios with various combinations of frictions are artificially created by varying *T*_*c*_ and *B* components in (8). The selection of *C* is performed offline via the following steps:

(a) estimate the bounds of *T*_*c*_ and *B* based on experimental studies.
(b) Deploy *K*_*d*_(0) in SIMULINK and find the corresponding *e*(0) over a certain number of cycles (say 15).
(c) Determine Δ*K*_*d*_(1) = *C* · *e*(0), using systematically increasing *C* values (starting from zero), so that the resulting error *e*(1) becomes desirably small (e.g., 75% as we used in our tests) compared with *e*(0) under most adverse friction conditions. At the same time, we note that *K*_*d*_ should be upper-bounded as it multiplies the encoder-based angular speed and tends to saturate the actuator input (*U* in Figure 4). Note that the determination of *C* becomes critical from the concern of minimizing the number of gain updates before the error converges to zero. Using the proposed method, determination of constant *C* is done offline.

The following portion is devoted to the convergence of *e* to zero and the determination of *K*_*d*_ over some experimental data. We give an interpretation of the earlier assigned variable *K*_*d*-ref_. “Reference *K*_*d*_, *K*_*d*-ref_” is the updated derivative gain which leads to smaller amplitude error |*e*_ref_| than that which evolves under the previous *K*_*d*-ref_. And the corresponding new error is denoted by *e*_ref_. To ensure that *e* converges to zero, the update law of *K*_*d*_ always moves the gain in the direction of smaller |*e*_ref_|. The update of *K*_*d*-ref_ is based on *e*_ref_ (i.e., Δ*K*_*d*_ = *C* · *e*_ref_). If the resultant |*e*| < |*e*_ref_|, this update results in a new *K*_*d*-ref_ and *e*_ref_. Otherwise, we can continue reducing this Δ*K*_*d*_ by 50% until a new *K*_*d*-ref_ as described in the updating sequence earlier. 

**Figure 7:**
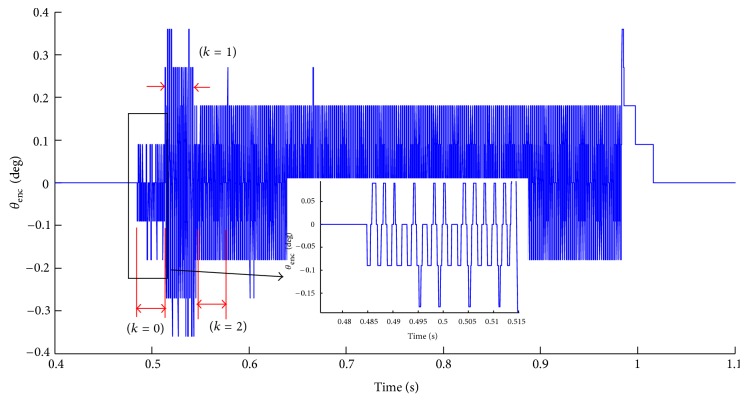
Experimental results with second adaptation scheme (*k* = 0,1, 2 refer to the stages in Table 1).

**Figure 8:**
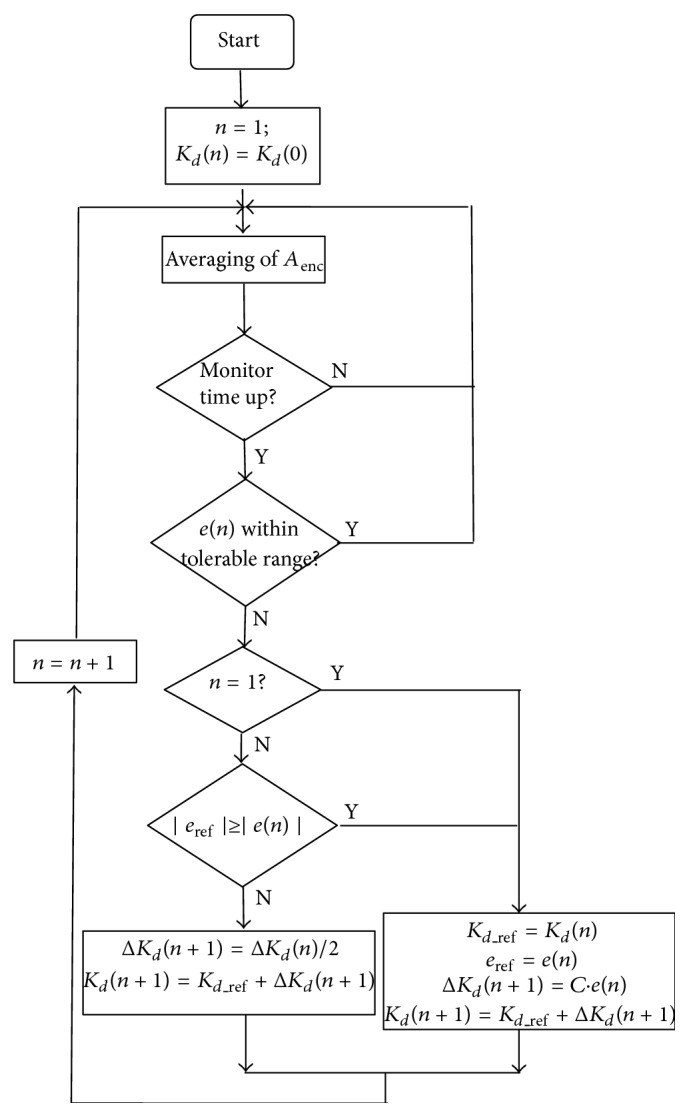
Flowchart of monitoring and on-line tuning mechanism.

This update process of *K*_*d*_ is shown over an example experimental case study. In this case, experiment is done on our Ros-Drill setup for the desired amplitude of 0.2 degrees and frequency of 500 Hz. The update of *K*_*d*_ is performed every 15 oscillations and throughout *D* (i.e., 500 ms).  Figure 7 shows the experimental result. Following the logic above, *C* = 0.0256 is selected for the present operating conditions. The microcontroller is programmed to monitor the average peak-to-peak value of *θ*_enc_ over 15 oscillations (i.e., $\bar{A_{\text{enc}}}$).  Table 1 shows the update process of *K*_*d*_ for this experiment. In the first round of tuning (marked as *k* = 0 in Table 1 and Figure 7), *K*_*d*_(0) = 0.0036 is selected from the look-up table for operating frequency of 500 Hz. However, *e*(0) = 1.9Δ is large (see the inset in Figure 7), meaning that the peak-to-peak strokes are much smaller than 4Δ. In the second round of tuning (*k* = 1 in Table 1 and Figure 7), *C* · *e*(0) is used to update *K*_*d*_ and *K*_*d*_(1) = *K*_*d*-ref_ + *C* · *e*(0) = 0.008. Because *K*_*d*_(1) results in |*e*(1)| > |*e*_ref_|, Δ*K*_*d*_(1) is bisected for the third round of tuning (*k* = 2 in Table 1 and Figure 7), *K*_*d*_(2) = *K*_*d*-ref_ + Δ*K*_*d*_(1)/2. Because |*e*(2)| < |*e*_ref_|, *K*_*d*_(2) and *e*(2) are taken as new *K*_*d*-ref_ and *e*_ref_, respectively. Tuning updating process terminates here. The controlled oscillation of pipette lasts for a predetermined duration and then the pipette is returned to starting “zero position” in order to prevent the wrap-around effect (on the tubing attachments). In Figure 7, at 0.98 sec, oscillation of pipette ends and begins to return to “zero position.” Figure 8 shows the flowchart of this monitoring and on-line tuning mechanism.

The first part of the paper, which is related to the new, second-stage adaptation scheme, ends here. In what follows, we will demonstrate the influence mechanism for the actual angular motion, if the encoder reading of the stroke is *A*_enc_ = 4Δ just to provide a better insight to the reader. 

## 5. Comprehension of Actual Angular Motion from Coarse Encoder Readings

The above control scheme is designed to perform an adaptively updating logic to assure a given *A*_enc_ peak-to-peak stroke. Let us take *A*_enc_ = 4Δ for the sake of simplified arguments, without loss of generality. We query which operational conditions are satisfied if *A*_enc_ = 4Δ reading is guaranteed, next. This quest results in some interesting observations which are stated in this section. Note that the derivations pertaining to *A*_enc_ = 4Δ in what follows can be generalized with ease to cases when desired control performance is *A*_enc_ = 2*m*Δ, for *m* = 1,2,….

To clarify some definitions about encoder reading, we list them as follows:

(i) a duration of oscillations = *D*;
(ii) absolute deviation of *A*_12_ from its nearest odd integer multiple of Δ = *ε*.

A very important observation is stated next. One can see from Figure 3 that for the encoder to register 4-step stroke (i.e., *A*_enc_ = 4Δ), the following are the necessary and sufficient conditions:

(9a) $$\begin{array}{c} -a+2\Delta <A_{1}<-a+3\Delta , \end{array}$$

(9b) $$\begin{array}{c} a+\Delta <A_{2}<a+2\Delta . \end{array}$$

By summing (9a) and (9b), we obtain

(10) $$\begin{array}{c} 3\Delta <A_{12}<5\Delta . \end{array}$$

Here, we define *ε* as the absolute deviation of *A*_12_ from the nearest odd integer multiple of Δ and 0 < *ε* < Δ. For 3Δ < *A*_12_ < 5Δ, we have

(11a)  either  3Δ<A12<4Δ,  ε=A12−3Δ

(11b)  or  4Δ≤A12<5Δ,  ε=5Δ−A12.

For the simplicity of expressions, let us denote

(12) $$\begin{array}{c} \bar{A_{12\text{-}b}}=\frac{\Delta -2a}{2}. \end{array}$$

Note that the offset angle is an uniformly distributed random variable between zero and Δ. Hence, $\bar{A_{12\text{-}b}}$ is also an uniformly distributed random variable but between −Δ/2 and Δ/2.

**Proposition 1 .**

For any harmonic motion with (2*m* − 1)Δ < *A*_12_ < (2*m* + 1)Δ, peak-to-peak encoder reading will always be *A*_*enc*_ = 2*m*Δ if and only if the bias of stroke (*A*_12-*b*_) satisfies

(13) $$\begin{array}{c} -\frac{\varepsilon }{2}<A_{12\text{-}b}-\bar{A_{12\text{-}b}}<\frac{\varepsilon }{2}. \end{array}$$

Furthermore if $A_{12\text{-}b}\;=\;\bar{A_{12\text{-}b}}$, for such a harmonic motion the encoder reading will always be 2*m*Δ.

**Proof**

Without loss of generality, let us take *m* = 2 and prove the sufficiency of (13) for *A*_enc_ = 4Δ. By substituting (2) and (12) into (13), we obtain

(14a) $$\begin{array}{c} \Delta -2a-\varepsilon <A_{1}-A_{2}<\Delta -2a+\varepsilon . \end{array}$$

(a) First consider the interval 3Δ < *A*_12_ < 4Δ. Note from (11a):

(14b) $$\begin{array}{c} A_{12}=A_{1}+A_{2}=3\Delta +\varepsilon . \end{array}$$

Using (14a) and (14b) once for *A*_1_ and again for *A*_2_, we obtain

(15a) $$\begin{array}{c} -a+2\Delta <A_{1}<-a+2\Delta +\varepsilon , \end{array}$$

(15b) $$\begin{array}{c} a+\Delta <A_{2}<a+\Delta +\varepsilon . \end{array}$$

Keeping in mind that 0 < *ε* < Δ, one can see that satisfying (15a) and (15b) automatically satisfies (9a) and (9b), which are the necessary and sufficient conditions for *A*_enc_ = 4Δ measurement (as mentioned at the beginning of Section 5).

(b) One should next consider the interval 4Δ < *A*_12_ < 5Δ. Note from (11b):

(16) $$\begin{array}{c} A_{12}=A_{1}+A_{2}=5\Delta -\varepsilon . \end{array}$$

Using (14a) and (16) once for *A*_1_ and again for *A*_2_, we obtain

(17a) $$\begin{array}{c} -a+3\Delta -\varepsilon <A_{1}<-a+3\Delta , \end{array}$$

(17b) $$\begin{array}{c} a+2\Delta -\varepsilon <A_{2}<a+2\Delta . \end{array}$$

With the condition 0 < *ε* < Δ, (17a) and (17b) guarantee the fulfillment of (9a) and (9b), respectively. Again they are the necessary and sufficient conditions for *A*_enc_ = 4Δ measurement. This completes the proof of sufficiency of Proposition 1.

Next we handle the necessity clause of the proposition. We take into account that *A*_enc_ = 4Δ and, therefore, ((9a) and (9b)) conditions hold. We wish to show that this necessitates (13). Let us again consider 3Δ < *A*_12_ < 4Δ first. Under this assumption, the right inequalities of ((9a) and (9b)) are satisfied automatically. The left inequality of (9a) is −*a* + 2Δ < *A*_1_ and it can be expressed as

(18a) $$\begin{array}{c} -\left(A_{1}+A_{2}-3\Delta \right)<A_{1}-A_{2}-\left(\Delta -2a\right). \end{array}$$

Similarly, the left inequality of (9b) is *a* + Δ < *A*_2_ and it can be rewritten as

(18b) $$\begin{array}{c} A_{1}-A_{2}-\left(\Delta -2a\right)<A_{1}+A_{2}-3\Delta . \end{array}$$

Using the definitions in (2) and (12) and the constraint (14b), the combined inequalities of ((18a) and (18b)) render exactly the conditions given in (13).

Let us now focus on the interval 4Δ < *A*_12_ < 5Δ. We also claim that this condition, this time, together with the right inequalities of ((9a) and (9b)), will satisfy the left inequalities of ((9a) and (9b)). Following the similar procedure above, with the right inequality of (9a), *A*_1_ < −*a* + 3Δ, we arrive at

(19a) $$\begin{array}{c} A_{1}-A_{2}-\left(\Delta -2a\right)<5\Delta -A_{1}-A_{2}. \end{array}$$

The right inequality of (9b), *A*_2_ < *a* + 2Δ, creates

(19b) $$\begin{array}{c} -\left(5\Delta -A_{1}-A_{2}\right)<A_{1}-A_{2}-\left(\Delta -2a\right). \end{array}$$

Once again, using the definitions in (2) and (12) and the constraint (16), the combined inequalities of ((19a) and (19b)) render exactly the conditions given in (13). This completes the necessity clause of the proposition.

Furthermore, if $A_{12\text{-}b}=\bar{A_{12\text{-}b}}$, (13) automatically holds for any *A*_12_ within 3Δ < *A*_12_ < 5Δ; therefore, the causality follows. This proof for *m* = 2 can be extended for cases when *A*_enc_ = 2*m*Δ, for *m* = 1,2,….

QED

The important implications of Proposition 1 can be summarized in the following logical sequences:

(i) $\bar{A_{12\text{-}b}}$ is a random number (due to the term “*a*”). However, “*a*” remains unchanged during the oscillatory period *D*; thus, $\bar{A_{12\text{-}b}}$ remains constant in the same period.
(ii) Bounds expressed by (13) suggest that the bias, *A*_12-*b*_, of each cycle during *D* stays within a bounded distance from $\bar{A_{12\text{-}b}}$  (i.e., *ε*/2) if and only if we maintain the encoder reading of *A*_enc_ = 2*m*Δ.
(iii) In essence, if a control mechanism which is described earlier assures the *A*_enc_ = 2*m*Δ, this also brings a guarantee for the boundedness of $\left|A_{12\text{-}b}-\bar{A_{12\text{-}b}}\right|<\varepsilon /2$, where *ε* is defined as (11a) and (11b). This implies that the bias *A*_12-*b*_ has an attractive bonding to $\bar{A_{12\text{-}b}}$. It can fluctuate around this value but cannot run away from it.

## 6. Conclusions

A novel control system with low-resolution encoder for the desired harmonic trajectory is studied on a cellular microinjection technology called the Ros-Drill (rotationally oscillating drill). In the first part of paper, a novel adaptive control logic is developed to facilitate the tracking of the harmonic rotational motion under uncertainties, especially frictions. We demonstrate via dynamics simulations first, followed by experiments that the tracking of the harmonic rotational motion is achieved with desirable fidelity. In the second part, a stochastic analysis connecting the actual motions and their low-resolution sensory recordings is presented. It is observed that when the control structure guarantees a fixed peak-to-peak stroke, the bias of actual angular motion is bounded in a range and it is attracted to a certain predefined value.

## Figures and Tables

**Table 1:** Update process of *K*_*d*_ for Figure 7.

*k*	*K*_*d*_(*k*)	*e*(*k*)	*e*_ref_	*K*_*d*-ref_	Δ*K*(*k* + 1)
0	0.0036	1.9Δ	1.9Δ	0.0036	*C* · *e*(0)
1	0.0080	−2.66Δ	1.9Δ	0.0036	Δ*K*_*d*_(1)/2
2	0.0058	0	0	0.0058	0

## Nomenclature

*θ*: Angular position of pipette holder (deg)
*θ*_enc_: Angular position sensed by encoder (deg)
*θ*_*d*_: Desired harmonic trajectory (deg)
*A*_*d*_: Amplitude of *θ*_*d*_ (deg)
*f*_*d*_: Frequency of *θ*_*d*_ (Hz)
*f*_*s*_: Control sampling frequency (Hz)
*K*_*p*_: Proportional control gain
*K*_*i*_: Integral control gain
*K*_*d*_: Derivative control gain
Δ: Resolution of position sensor
*a*: Encoder offset angle (deg)
*A*_1_: Upper amplitude of angular position (deg)
*A*_2_: Lower amplitude of angular position (deg)
*A*_12_: Peak-to-peak angular stroke of *θ* (deg)
*A*_12-*b*_: Bias of angular stroke (deg)
*A*_enc_: Peak-to-peak angular stroke of *θ*_enc_ (deg)
$\bar{A_{12}}:$: Average angular stroke over a certain number of oscillations (deg)
$\bar{A_{\text{enc}}}:$: Average encoder angular stroke over a certain number of oscillations (deg)
*D*: A duration of oscillations
*ε*: Absolute deviation of *A*_12_ from its nearest odd integer multiple of Δ
*U*: Voltage input to the micromotor (V)
*J*: Moment of inertia of the microinjector (g · cm^2^)
*L*: Armature inductance (*μ*H)
*R*: Armature resistance (Ω)
*K*_*e*_: Motor back EMF constant (mV/rpm)
*K*_*m*_: Motor torque constant (mNm/A)
*T*_*c*_: Coulomb friction torque (Nm)
*B*: Viscous friction coefficient (Nms/rad).
